# Advances in Controllable Release Essential Oil Microcapsules and Their Promising Applications

**DOI:** 10.3390/molecules28134979

**Published:** 2023-06-25

**Authors:** Yana Zhao, Yanbo Wang, Zhijun Zhang, Huizhen Li

**Affiliations:** School of Chemistry and Chemical Engineering, North University of China, Taiyuan 030051, China

**Keywords:** essential oils, microcapsules, controllable release, application

## Abstract

Essential oils (EOs) have emerged as natural and popular ingredients used in the preparation of safe and sustainable products because of their unique characteristics, such as antibacterial and antioxidant activity. However, due to their high volatility, poorly solubility in water, and susceptibility to degradation and oxidation, the application of EOs is greatly limited. One of the promising strategies for overcoming these restrictions is encapsulation, which involves in the entrapment of EOs inside biocompatible materials to utilize their controllable release and good bioavailability. In this review, the microencapsulation of the controllable release EOs and their applications are investigated. The focus is on the antimicrobial mechanism of various EOs on different bacteria and fungi, release mechanism of microencapsulated EOs, and preparation research progress of the controllable EOs microcapsules. In addition, their applications are introduced in relation to the food, textiles, agriculture, and medical fields.

## 1. Introduction

Essential oils (EOs) are secondary metabolites produced from plant tissues, such as flowers, buds, stems, leaves, roots, fruits, bark, resins, or wood, and they are mainly stored in secretory cells, epidermal cells, cavities, canals, or glandular trichomes of plants [1]. EOs are complex mixtures of odoriferous, volatile, and oily hydrophobic aromatic liquids. The main chemical composition includes monoterpenes, sesquiterpenes, diterpenes, and their oxygenated forms. Additionally, there are various phenols, aldehydes, ketones, esters, alcohols, and hydrocarbons present in EOs, such as eugenol [2], carvacrol [3], and limonene, [4] etc., which impart to them broad biological activity, such as antioxidant, antimicrobial, insecticidal, and therapeutic properties. Currently, approximately 3000 EOs have been established, 300 of which are especially important in the food, medicine, textile, cosmetics, and agricultural fields [5].

**Figure 1:**
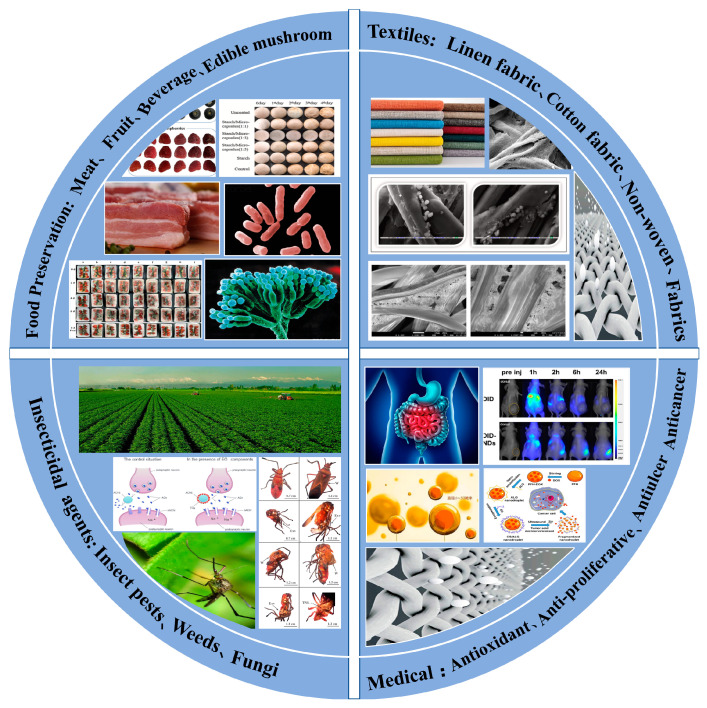
Application of EOs microcapsules in food, textiles, agriculture, and medical fields.

EOs are considered highly valuable rare substances owing to their low extraction yields (approximately 1%). Additionally, EOs are extremely unstable, and several external factors (e.g., oxygen, light, acidic or alkaline conditions, etc.) can lead to the degradation, transformation, or volatilization of EOs and reduce their biological activity [6]. Microencapsulation technology involves embedding active substances in wall materials, protecting core materials from environmental influence and denaturation, and achieving controllable release or stable storage of active substances [7]. Thus, the microencapsulation of EOs is very crucial to protecting them from the effects of adverse conditions and ensure their quality. Usually, microcapsules are formed by natural or synthetic polymer materials through various interactions during the preparation process, such as ion interaction, electrostatic interaction, hydrophilic hydrophobic interaction, covalent bonds, and hydrogen bonds. According to the preparation principles, the preparation methods of microcapsules can be divided into physical methods (e.g., interfacial polymerization, inclusion complexation, ionic gelation, coacervation, etc.) and chemical methods (e.g., spray-drying method, lyophilization, emulsification method, oil/water method, ultrasonic method, solvent evaporation method, phase dispersion coacervation method, layer-by-layer assembly method, layer-by-layer desolvation method, etc.) [8,9]. Generally, more than one of the above methods are often combined to prepare the expected microcapsules. Appropriate preparation methods can improve the encapsulation efficiency of EOs and reduce economic consumption. Many authors in previous investigations have confirmed the strong influences of encapsulation methods, total solid content, emulsions properties, and interactions between EOs and wall materials on encapsulation efficiency [10,11]. According to the research of Mehran et al. [12] and Jafari et al. [13], encapsulation efficiency increases with an increase in total content of wall materials. The results can be related to the increase of emulsion viscosity. In addition, the higher temperature of spray drying has a negative effect on encapsulation efficiency. This outcome can be attributed to the breakdown of the microcapsule wall at higher temperatures, consequently resulting in the release of EOs. In this review, we summarize the antimicrobial mechanism of various EOs on different bacteria and fungi as well as the release mechanism and preparation research progress of controllable EOs microcapsules with various natural and stimuli-responsive substances as wall materials. In addition, the applications of controllable release EOs microcapsules are introduced into the fields of food, textiles, agriculture, and medicine (Figure 1).

## 2. Antimicrobial Mechanism of EOs

Most EOs have a wide spectrum of antimicrobial activity against foodborne pathogenic and spoilage microorganisms, and the antimicrobial capacity is largely dependent on their chemical constituent and cell type. For example, trans-cinnamaldehyde is one of the major compounds of EOs and has the proficiency to inhibit the growth of *E. coli* by depleting intracellular ATP levels, reducing bacterial cell division protein FtsZ expression, GTP-dependent polymerization, and GTP hydrolysis [14]. Phenolic compounds, such as carvacrol, eugenol, and thymol, can effectively inhibit the growth of microorganisms by disrupting cell membranes, leading to alteration in electron flow, driving force of protons and active transport, and coagulation of cell contents [15,16]. Additionally, thymol is also able to inhibit the expression of thioredoxin-1 and is effective against several vital enzyme pathways and metabolites, such as the phosphotransferase system, the efflux system, and energy metabolism. Monoterpenes target membrane-embedded proteins, membrane fluidity–permeability mechanisms, and ion transport processes [17]. Carvone is another important EO constituent which can disrupt the outer membranes of the cells but does not affect cellular ATP pools [18]. In general, the major compounds are largely responsible for antibacterial activity of different EOs, and this activity is attributed to the free hydroxyl groups of these compounds. EOs containing aldehyde or phenolic groups, such as cinnamaldehyde, linanaldehyde, eugenol, and thymol, have shown the highest antibacterial activity, followed by EOs containing terpenes (e.g., β-myrcene, α-thujone, etc.), which exhibit weaker antibacterial effects [19]. Other EOs containing ketones or esters have much weaker activity, and EOs containing terpene hydrocarbons are usually inactive [20,21].

Owing to the different compositions and thicknesses of cell membranes, EOs display varying resistances against different types of microorganisms. Most components (90–95%) in the cell wall of gram-positive bacteria are peptidoglycan, which allows easy diffusion of hydrophobic components, such as EOs, into cells and can directly damage cell membranes, block enzyme systems, and increase ion permeability. In contrast, the peptidoglycan layer is thinner for the cell walls of gram-negative bacteria, which are covered by outer membranes that consisting of double-layer phospholipids. The hydrophilic transmembrane channels at the surface of the outer membrane leads to the easy penetration of hydrophilic substances, while the hydrophobic molecules, such as EOs, slowly traverse the outer membrane through porins due to the interaction between EOs and the lipopolysaccharide layer [22,23]. Therefore, the antibacterial effects of EOs against gram-positive bacteria are reported to be higher than against gram-negative bacteria. For example, *cunila* EOs display more pronounced antimicrobial activity against gram-positive bacteria than against gram-negative bacteria [24]. However, eugenol and isoeugenol are more active against gram-negative bacteria compared to gram-positive bacteria [25].

EOs can easily penetrate the lipid layer of the cell wall and disrupt the cell membrane’s structure, thereby exerting multiple antibacterial mechanisms through action on intra-cellular proteins, enzymes, DNA, metabolism, energy production, etc. In this review, the inhibitory mechanisms of different EOs on various bacteria (Table 1) and fungus (Table 2) are revealed from the aspects of the biomacromolecules, such as DNA, proteins, and lipids; the cell wall or cell membrane integrity; the metabolic pathway; and the expression of related virulence genes.

## 3. Preparation of Controllable-Release EOs Microcapsules

Currently, in terms of wall materials, EO microcapsules include single-layer microcapsules, multilayer microcapsules, and stimuli-responsive microcapsules. Generally speaking, the release of microcapsules can be divided into two stages, namely immediate triggered release, which is controlled by the start of the release system, and sustained release, which is mainly the continuous and one-time diffusion of EOs from the polymer shell before and after its rupture [50]. Different release mechanisms of the microencapsulated EOs have diffusion, fragmentation, polymer relaxation (shrinking/swelling), and erosion, which are substantially related to the type of wall materials and encapsulation method [51]. Additionally, it is very significant for the application of microcapsules. Table 3 summarizes the wall material types, microcapsule methods, release models, and release mechanisms of different EOs microcapsules.

### 3.1. Single-Layer Microcapsules

A wide range of substances, including polysaccharides (e.g., gum arabic, persian gum, maltodextrin, cyclodextrin, hydroxypropyl-β-cyclodextrin, inulin, modified starch, pectin, sodium alginate, and chitosan, etc.), proteins (e.g., gelatin, whey protein, mung bean isolate protein, rice protein, zein, etc.), lipids, poly (D, L-lactide-co-glycolide) (e.g., polyvinyl alcohol, polyurethane, etc.), and silica-biocompatible materials are some of the wall materials used in the food and medical fields. Especially for plant-derived proteins/polysaccharides, all exhibit good biocompatibility, biodegradability, bioadhesion, and non-toxicity and have been widely applied for food processing due to their advantages of lower cost, higher sustainability, and versatility [63,64]. Dima et al. [52] has prepared coriander EO microcapsules by spray-drying with chitosan, alginate, chitosan/alginate, or chitosan/inulin as wall materials. The release kinetics show that the swelling degree and release rate of chitosan microcapsules are the highest at pH 2.5, and that those of alginate microcapsules are the highest at pH 6.5 in the simulated human gastrointestinal digestive system. However, they are all resistant to pH and temperature variations and can ensure a slow release of coriander EO. Das et al. [65] and Hasheminejad et al. [66] have encapsulated *Coriandrum sativum* EO and clove EO with chitosan-based nanomatrix, which is able to improve the shelf life of stored rice and pomegranate arils and is obviously better than free EOs. However, owing to the different polarities of EOs and chitosan, the binding force is low and the chitosan-containing EOs rapidly lose their antimicrobial properties, resulting in insufficient efficacy [67]. Bajac et al. [54] encapsulates juniper berry EO by spray-drying with gum arabic, maltodextrin, sodium alginate, or whey protein concentrate as wall materials. The release kinetics indicate that the release time and speed of the microcapsules formed by sodium alginate (almost all release within 15 min) are faster than those of gum arabic (almost all release within 30 min). However, using a combination of maltodextrin and gum arabic as wall materials allows the slowest release from microparticles, and it further prolongs the complete release of EOs to about 45 min. An antimicrobial sachet releasing vapor of oregano EO is developed by spray-drying using polyvinyl alcohol as wall material, and release of oregano EO occurs under conditions of high humidity and room temperature [68]. Traditional single-layer encapsulation has the disadvantages of poor mechanical and barrier properties. Therefore, multilayer encapsulation has attracted considerable attention in recent years.

### 3.2. Multilayer Microcapsules

Multilayer encapsulation is a method of achieving sustained release of EOs using multiple wall materials, which prepares by “layer-by-layer” (LBL) self-assembly technology, involving electrostatic deposition between wall materials with opposite charges and spontaneous crosslinking to form a polyelectrolyte layer. Multilayer encapsulation uses EOs as the intermediate or inner layer, which can effectively improve slow release and retention rate of EOs [69]. LBL technology mainly focuses on the homogeneous emulsification of mixtures. The preparation process should be such that the instability caused by bridging, consumption, flocculation, or coalescence of the substrate is avoided. Simultaneously, the size distribution, shell composition, shell porosity, and diffusion of the condensed layer affect EO release [70]. Composite coacervation has become the most commonly used multilayer encapsulation method owing to its high stability and loading rate, which is a polymerization phenomenon initiated by two biopolymers with opposite charges—generally proteins (amino) and polysaccharides (sugar carboxyl)—through electrostatic forces, hydrogen bonds, or hydrophobic forces under strict control of process parameters such as pH, temperature, ionic strength, stirring rate, polymer ratio, concentration, and molecular weight [71].

Qiu et al. [64] prepares double-layer microcapsules containing mung bean isolated protein/apricot peel pectin/rose EO. The microcapsules release rose EO rapidly for the first 9 min and then release rose EO continuously over the next 26 min. β-Lactoglobulin/sodium alginate/black pepper EO microcapsules give similar results and are very stable in the in vitro digestion (oral and gastric) stage, in which 65.5% of EO is transported to intestine. Mehran et al. [12] used a spray-drying method to prepare inulin/gum arabic/peppermint EO microcapsules. The system explosively releases EO within 7 min of the start, and then the release rate decreases successively in 50% ethanol, 10% ethanol, 3% acetic acid, and distilled water. The Peppas–Sahlin model (Fickian diffusion) is the best kinetic model for describing the release of peppermint EO. Cyclodextrins have hydrophilic surfaces and hydrophobic cavities. Natural cyclodextrins, including α-, β-, and γ-cyclodextrins, are widely used because of their suitable cavity sizes, high retention rates, and low price. Compared to β-cyclodextrin, hydroxypropyl-β-cyclodextrin has higher water solubility and lower toxicity and has been approved by the FDA for use as food additives. Hou et al. [72] prepared a hydroxypropyl-β-cyclodextrin/cinnamon EO nanoemulsion which can protect EOs and enable sustained release to exert long-term antibacterial effects. An EO viscous core microcapsule is formulated by encapsulating EOs with intermediate non-crosslinked polymer shells and crosslinked polymer outer shells followed by dissolution of the intermediate shells with EOs to form viscous cores. The study found that the microcapsule has better heat resistance, extreme long-lasting release property, and viscoelasticity and that it is more adhesive to cotton fabrics, which is more consumer-friendly compared to melamine-formaldehyde microcapsules [58]. According to the research of Noghabi et al. [59], maltodextrin is used in combination with Persian gum to achieve proper the encapsulation of cinnamon EO by spray drying. Maltodextrin as secondary wall material protects cinnamon EO against oxidation appropriately and offers low viscosity at high solids concentration. Sun et al. [73] has used porous starch as a core material carrier to adsorb fennel EO and sodium alginate/chitosan as wall materials to successfully prepare sodium alginate/chitosan/porous starch–fennel EO microcapsules using a polyelectrolyte composite coagulation method. In the open and closed systems, the cumulative release rates of fennel EO in 16 days are 70.62% and 43.87%, respectively, with a good slow-release effect. Basil EO microcapsules are successfully produced with different wall materials, including gum arabic, maltodextrin, and whey protein isolate. Results show that the ternary combination has a high product yield, encapsulation efficiency, and eugenol release rate in ethanol [74]. A star anise EO microcapsule was produced from rice protein and depolymerized pectin using electrostatic complexation and exhibits a sustained release and good antibacterial effect [75]. Compared to single-layer encapsulation, multilayer microcapsules have higher stability and resistance to environmental strains, such as oxidation, thermal change, and ionic strength, which can effectively improve the physical and chemical stability of EOs and allow the control and triggering of the retention and release characteristics of EOs, achieving continuous, high-content, and uniform release [76].

### 3.3. Stimuli-Responsive Microcapsules

With the continuous development of microencapsulation technology, the function of embedding and protecting core materials of traditional microencapsuation can no longer meet the needs of industry. Recently, more and more scholars have paid attention to targeted, controllable, triggered, and other special functional releases. Stimuli-responsive microcapsules have drawn great attention in various fields and are involved in the permeability or rupture changes of wall materials under specific environmental conditions, such as temperature, humidity, pH, light, enzymes, magnetic fields, etc., which cause the EOs to be released. These environmental-stimuli-responsive microcapsules have the advantages of rapid response, large internal capacity, and stable capsule membrane [77]. EOs can maintain high activity for a given time and continuously produce activity where it is needed. Chitosan, poly (D, L-lactide-co-glycolide), protein fragment (gelatin), and silica biocompatible materials are some of the wall materials used in the stimuli-responsive microcapsules field [78]. Xu et al. [57] has established a triggering system of core-shell structure, oregano EO loaded microcapsules using chitosan-decorated TiO_2_ (a UV-light-sensitive shell material) as wall materials, which can be used to control the release by UV irradiation depending on specific needs and can be applied in the food industry. Compared with single-shell or single-stimulated microcapsules, the controllable release of core substances from double-shell microcapsules is more durable. The outer shell not only provides a good and stable environment for the inner layer, but the design of double-shell layer structure can also make the layers independent from each other, and the improvement of different layers can provide multiple functions to the microcapsules. Zhu et al. [55] prepared a bio-based, material-modified polylactic acid/octenyl succinic anhydride–chitosan/tea tree EO microcapsule with a pH-responsive function and antibacterial properties via the double emulsion method and the solvent evaporation method, which can accelerate the release at pH = 5 and maintain 100% antibacterial efficiency after 72 h. A new double-shell lignin microcapsule containing lemon EO was successfully fabricated using the interfacial polymerization and free radical polymerization methods. Lemon EO is further located at the core of the microcapsule and is released by thermal response [60]. As natural biocompatible polysaccharides, anionic sodium alginate and cationic chitosan can be widely used to prepare various drug delivery systems owing to their pH sensitivity and enzymatic degradability, which can achieve the selected release of EOs in a specific environment [79].

## 4. Application of Controllable Release EOs Microcapsules

### 4.1. Food Industry

Foodborne spoilage and pathogenic bacteria are major concerns contributing to food quality and safety, with foodborne diseases constituting a global health problem. Chemical antisepsis is an important method adopted by the food industry to control foodborne microorganisms and prolong food shelf life. However, in recent years, owing to food safety accidents caused by synthetic preservatives and higher requirements for food quality and flavor, the development of natural, environmental, and healthy preservatives has become a new trend. EOs derived from plants have become a promising natural antiseptic candidate owing to their powerful and broad-spectrum antibacterial activity. An appropriate amount of EOs does not affect the flavor of the product and increases the appetite of consumers to a certain extent. Additionally, EOs have been generally recognized as safe (GRAS), and some are approved by the U.S. Food and Drug Administration (FDA) to be used as food additives, such as lemon, basil, coriander, clove, thyme, and vanilla EO. Currently, approximately 3000 of EOs are known, and some of them are commercially important, being employed in the agronomic, cosmetic, chemical, perfume, fragrance, pharmaceutical, and food industries [80,81].

Research indicates that the interaction of polysaccharides and proteins could improve the stability of protein nanoparticles to the environment. Cai et al. [82] prepared a multilayer encapsulated gliadin/glycyrrhizic polysaccharide/tea tree EO nanoparticle. The inhibition rates of the EOs nanoparticles on *Salmonella typhimurium* on the surface of pork and chicken products were 98.52% and 97.86%, respectively. At the same time, the EO nanoparticles delayed lipid peroxidation of meat products, and the quality and color of meat products did not change significantly within the 5-day storage period. Recently, various microcapsules loaded with EOs have been used for active film/coating applications. For example, a microcapsule loaded with oregano EO via ionic gelation has been developed. Sodium alginate is used as the wall material, with calcium chloride as a crosslinking agent. Compared to soy protein concentrate films with free oregano EO, incorporating the oregano EO microcapsule significantly improves the composite film’s antimicrobial ability against *E. coli* and *S. aureus* [83]. Additionally, EOs can be used to develop biodegradable active packaging, which is an interesting substitute in the food packaging industry. To improve the slow-release effect of cinnamon EO, β-cyclodextrin/cinnamon EO microcapsules were firstly prepared by Shao et al. [84]. Continuously, the starch/cinnamon EO microcapsules were used as coating for the paper to prepare bioactive packaging materials, which had remarkable bacteriostatic effects on mesophilic, psychrophilic, pseudomonad, yeast, and mold counts. This can effectively prolong the storage life and ensure the safety of mushrooms. Modified atmosphere packaging is considered a good method to help maintain post-harvest quality and prolong the storage time of fruit. Li et al. [85] prepared the 3D printing of lemongrass EO/β-cyclodextrin/popping candy modified atmosphere packaging for strawberry preservation. Lemongrass EO was successfully encapsulated in β-cyclodextrin and had a strong bacteriostatic effect on *Aspergillus niger* and *Botrytis cinerea*. Simultaneously, the popping candy can release CO_2_ and extend the shelf life of strawberries. Tian et al. [86] firstly reported the preparation of sustained-release tea tree EO solid preservative based on polysaccharide. The hydrogen bond interaction of the starch–carboxymethyl cellulose network structure and the hydrophobic interaction of SiO_2_-adsorbing tea tree EO resulted in a compact structure and excellent sustained-release performance of the solid preservative, which improved sensory quality and reduced nutrient loss and microbial spoilage (molds and yeasts) in modified atmosphere packaging. Biodegradable and effective starch-based anti-bacterial film packagings have been successfully made by incorporating polyurethane-encapsulated EO microcapsules, including tree EO microcapsules, lavender EO microcapsules, and perilla leaf EO microcapsules as alternative synthetic preservatives for food preservation [87].

### 4.2. Textiles

Textiles are one of the most important necessities in daily life. With the increasing demand of consumers for disinfected and easy-care textiles, and the continuous improvement of people’s health awareness, the production of multifunctional medical natural textiles with antibacterial, antioxidant, insect-resistant, and sanitary properties has become a primary demand of customers. Owing to the adverse effects of many synthetic antibacterial agents and insect repellents on the environment and human health, EOs are being used as natural antibacterials and insect repellents due to their extensive biological activities in textile production [88]. Microencapsulation technology not only prevents EOs from leaking but also extends the antibacterial time and service life of the fabric coatings, and the release of the core material is controllable. A chitosan/gelatin/rosemary EO microcapsule has been utilized for the development of multifunctional and efficient linen fabric with a dip-rolling drying method. When the encapsulation efficiency of rosemary EO exceeds 70%, the multifunctional textile has long-lasting mosquito repellent ability (>90%), inhibition activity against *E. coli* (>93%) and *S. aureus* (>95%), significant anti-oxidant ability (>91%), and a pleasant aroma [56]. A gum arabic/citronella EO microcapsule prepared using a two-step method of emulsification and spray-drying has the advantages of prolonging the volatility and reducing the irritation of EOs, which can be dipped on non-woven fabrics for the preparation of cosmetic textiles [89]. A gelatin/gum arabic/citronella EO microcapsule is prepared through composite coagulation and spray-drying, and then they dip the microcapsules slurry into cotton fabric to prepare insect repellent textiles with the traditional dip-rolling drying method. The insect repellent effect within three weeks is more than 90%, which is a durable multifunctional textile [90]. Liu et al. [91] utilized chitosan–gelatin as a shell material to prepare microcapsules containing patchouli oil via the complex coacervation method, and the prepared microcapsules were grafted onto cotton fabric. The developed fabric displays 65% antibacterial activity for *S. aureus* and *E. coli* after 25 washes. Chen et al. [92] proposed a facile and environmentally friendly method for the preparation of aqueous multifunctional fabric coating using cellulose/silica hybrid microcapsules. They first synthesized lavender-fragrance-oil-loaded (LFO) cellulose/silica hybrid microcapsules containing EOs in one step through emulsion solvent diffusion and then added aqueous polysiloxane resins to prepare multifunctional fabric coating. The obtained fabric coating allows for pH-controllable stimulation of the release of LFO. The fabrics treated with the coating retained more than 30% of their fragrance after 90 days and exhibited excellent UV resistance. Exploring new combinations of biopolymers and EOs microcapsules for the development of multifunctional textiles is a broad prospect.

### 4.3. Agriculture Field

Chemical pesticides are severely restricted owing to serious negative consequences, such as soil and water contamination, high persistence in the environment and as residues in foods, and toxicity to people and animals. Therefore, plant pesticides—as effective, natural, and ecological insecticidal agents—can be developed and applied for integrated pest control. In general, EOs and their constituents can exhibit strong pesticide potential against insect pests, weeds, and fungi. Reports show that the neurotoxic effects of EOs can be prevented by blocking the octopamine receptors or by acetyl-cholinesterase inhibition and also through blocking the γ-aminobutyric acid receptor [93]. A gum arabic/maltodextrin/*Schinus molle* EO microcapsule has been employed to control the release of EO, which not only protects them from the external environment but improves insecticidal potential on *Haematobia irritans*. Compared to free EOs (96% mortality rate of flies within 2 h), the microcapsules have a slow-release effect (71% of EOs retention within 366 h) and a lasting insecticidal effect (32% and 73% mortality rate of flies within 2 h and 4 h, respectively) [94]. Similarly, Ahsaei et al. [95] prepared octenyl succinic anhydride–starch/*Rosmarinus officinalis* or *Zataria multiflora* EOs microcapsules using the spray-drying method, which displayed a more effective and long-term fight against *Tribolium confusum* than free EOs. Free EOs had no insecticidal activity after 15 days of storage, whereas the insecticidal rates of EOs microcapsules in the same period were 46.6% and 35.5%, respectively, indicating that microencapsulation increases the insecticidal persistence of EOs. Lv et al. [96] developed a novel, efficient, and safe antimildew for peanut kernel postharvest storage. The antimildew, cinnamon–*Litsea* cubeba compound essential oil (CLCEO) microcapsule was synthesized with CLCEO as the core material, and β-cyclodextrin was used as the wall material. CLCEO microcapsules can effectively reduce the total number of fungal colonies, the relative abundance of *Aspergillus* spp., and the atoxin B1 content of peanut kernels and have a positive effect on slowing down the increase in the acid value of peanut oil without causing any adverse effect on the viability and sensory properties during storage process. Thus, plant EOs have been explored as low-risk insecticides, some of which are classified under the Generally Recognized as Safe (GRAS) category and meet the criteria of reduced-risk pesticides [97].

### 4.4. Medical Field

EOs possess a number of beneficial biological properties, such as antioxidant, antimicrobial, anti-proliferative, antiulcer, anti-inflammatory, and anticancer activities. Reports show that the absorption of EOs mainly occurs in the distal small intestine rather than the stomach and proximal small intestine. Excessive and early release of EOs in the stomach has adverse side effects on human health, such as nausea, dizziness, and accelerated heartbeat. In this respect, microencapsulation may be an approach to achieving the protection and controllable release of EOs. Beeswax is a gastro-resistant material which is frequently used in the preparation of controllable release drug delivery systems. Beeswax microcapsules retard the release of water-insoluble drugs in the simulated gastric solution at pH 1.2 due to their highly crystalline structure and highly hydrophobic nature. A beeswax/carboxymethyl cellulose/gelatin holy basil EOs microcapsule was prepared using a simple coacervation method by Ngamekaue et al. [98]. It exposed the minimized holy basil EOs in simulated gastric fluids and delivered the EOs to the distal small intestine, displaying effective antioxidant and antibacterial activities. As natural biocompatible polysaccharides, anionic sodium alginate and cationic chitosan can be widely used to prepare various drug delivery systems owing to their pH sensitivity and enzymatic degradability, which can achieve the selected release of EOs based on the gastrointestinal microenvironment [79]. For example, a porous alginate/chitosan/basil oil microcapsule which retains the antioxidant and antimicrobial activities of basil oil under storage, in addition to a great tolerance to acids, bile, trypsin, and thermal conditions, was prepared [99]. However, the microencapsulation technique for EOs with effective and efficient delivery in animal’s bodies is a point to be further developed.

## 5. Conclusions and Prospect

EOs have been recognized as important natural sources with a widespread range of bioactivity. Considerable research reports the great prospects for EOs as bioactive ingredients in the food industry, pharmaceutical cosmetics, and botanical pesticide production. However, conventional formulation procedure will diminish EOs’ efficiency and limit their uses in practice owing to sensitivity. Thus, loading them in protective biopolymer materials will offer controllable release and delay their fast evaporation and degradation. In this review, we summarize the antimicrobial mechanism, the release mechanism of microencapsulated EOs, and the various natural and stimuli-responsive materials used for the fabrication of EOs microcapsules. In addition, the applications of controllable release EOs microcapsules are introduced in relation to the food, textile, agriculture, and medical fields.

However, the key technology now lies in manufacturing intelligent microcapsules with multiple reactions and compartments which can simultaneously respond to a wide range of stimuli, truly achieving intelligence. Additionally, attention should also be paid to enhancing the retention of active ingredients in microcapsules, especially when the microcapsules are stimulated by external environments. Future research may also use several innovative techniques to develop novel sustained EO release and retention enhancement strategies in microcapsules, such as low-temperature plasma, pulsed light, electrostatic self-assembly techniques, radiation, and electrospinning and electrospraying technologies. In addition, given the rising interest in EOs, consumer health concerns must be considered an urgent topic to be addressed. Although several organizations have collaborated with governments to implement the regulation of EOs worldwide, strong national and international regulations should be established before applying EOs microcapsules in various fields.

## Figures and Tables

**Table 1:** The inhibition mechanism of different essential oils (EOs) against various bacteria.

EOs	Bacteria	Antibacterial Mechanism
Clove EOs [26]	*Listeria monocytogenes*	(1) Biomacromolecules: change DNA structure [26]; (2) Cell wall/membrane integrity: destroy the integrity of the cell membrane, leading to the efflux of biomacromolecules [26].
Clove EOs [26] *Eugenia stipitata* EOs [27] Thyme EOs [28] Chuzhou chrysanthemum EOs [29] *Dodartia orientalis* L. EOs [30] Peppermint EOs [31]	*Staphylococcus aureus*	(1) Biomacromolecules: interact with DNA and affects the accessory gene regulator (Agr) system, resulting in decreased expression of virulence genes [26]; (2) Cell wall/membrane integrity: Disruption of cell membrane structure leading to the extravasation of ions (K^+^), nucleic acids, and proteins [27,29,30]; involves membrane potential depolarization and fluidity reduction possibly related to changes in fatty acid composition [28]; causes the destruction of structures and the function of the cell wall [30]; (3) Metabolic pathway: inhibits the respiratory metabolism by inhibiting the tricarboxylic acid cycle pathway [26]; affects Embden–Meyerhof–Parnas pathway and decreases key enzyme activity (PFK, HK and PK) [29]; improves the reactive oxygen species (ROS) and malondialdehyde (MDA) level [31].
*Lindera glauca* fruit EOs [32]	*Shigella* *flexneri*	(1) Cell wall/membrane integrity: disruption of cell membrane structure leading to the extravasation of ions (K^+^), nucleic acids, and proteins [32]; (2) Metabolic pathway: induces H_2_O_2_ accumulation and lipid peroxidation (ROS and MDA); inhibits respiratory metabolism and gives rise to a disturbance of redox homeostasis [32].
Chuzhou chrysanthemum EOs [29] *Dodartia orientalis* L. EOs [30] Peppermint EOs [31] *Alpinia galanga* rhizomes EOs [33] Thyme EOs [34,35] *Litsea cubeba* EOs [36]	*Escherichia* *coli*	(1) Biomacromolecules: disrupt DNA replication [36]; (2) Cell wall/membrane integrity: disruption of cell membrane structure leading to the extravasation of ions (K^+^), nucleic acids and proteins [28,30,33,35,36]; involving membrane potential depolarization and fluidity reduction possibly related to the changes of fatty acid composition [28]; causes the destruction of structure and function of cell wall [30]; (3) Metabolic pathway: affects hexose monophophate (HMP) pathway and decreases key enzyme activity (G6PHD) [29]; improves reactive oxygen species (ROS) and malondialdehyde (MDA) levels [31]; inhibits respiratory metabolism [36]; (4) Virulence gene expression: inhibits enzymatic activity (P-type ATPases) and down-regulates the expression of four virulence genes (stx1, stx2, ehxA, eae) [33,36].
*Dodartia orientalis* L. EOs [30] *Origanum vulgare* EOs [34]	*Salmonella Enteritidis*	(1) Cell wall/membrane integrity: disruption of cell membrane structure leading to the extravasation of ions (K^+^), nucleic acids, and proteins; causes the destruction of structure and function of cell wall [30]; (2) Biomacromolecules/metabolic pathway: DNA inhibition; key protein expression changes; bacterial oxidative stress; cellular metabolic imbalance [34].
Oregano EOs [37] *Litsea cubeba* EOs [38]	Methicillin-resistant *Staphylococcus aureus*	(1) Biomacromolecules: disrupts DNA replication by reacting with DNA in the form of chimera [38]; (2) Cell wall/membrane integrity: disruption of cell membrane structure leading to the extravasation of ions (K^+^), nucleic acids, and proteins [37,38]; (3) Metabolic level: inhibits respiratory metabolism by inhibiting the tricarboxylic acid cycle pathway and key enzymes [37]; affects HMP pathway and decreases key enzyme activity (G6PHD) [38]; (4) Gene expression: inhibition of relative expression of pvl gene [37,38].
*Artemisia asiatica* EOs [39]	*Haemophilus influenzae*	(1) Cell wall/membrane integrity: leads to cell wall damage, cell deformation, and cell shrinkage [39];
*Juniperus rigida* EOs [40]	*Klebsiella pneumoniae*	(1) Cell wall/membrane integrity: causes irreversible damage to the cell wall and membrane, leading to the leakage of proteins and DNA/RNA. [40];
Clove EOs [41]	*Burkholderia gladioli*	(1) Cell wall/membrane integrity: damages cell membranes, resulting in leakage of bacterial cytoplasm [41]; (2) Toxin expression: reduces the expression of bongkrekic acid [41];

**Table 2:** The inhibition mechanism of different EOs against various fungus.

EOs	Fungus	Antifungal Mechanism
*Chrysanthemum morifolium* cv. Fubaiju EOs [42]	*C. albicans*; *C. glabrata*; *C. tropicalis*; *S. cerevisiae*; *D. hansenii*; *Z. parabailii*	(1) Biomacromolecules: mitochondrial and DNA damage [42]; (2) Cell wall/membrane integrity: plasma membrane disruption [42].
Cinnamon EOs [43]	*Mucor* sp. FJ09; *Mucor circinelloides* CNRMA 03.0371	(1) Spore/mycelial: destroys the mycelial morphology [43]; (2) Cell wall/membrane integrity: increases membrane permeability, leading to the leakage of cellular components [43]. (3) Metabolic level: increases reactive oxygen species content and alters soluble sugar and malondialdehyde content [43]; (4) Gene expression: upregulation expression of nox1 and nox2 genes [43];
*Perilla frutescens* EOs [44] *Foeniculum vulgare* EOs [45]	*Aspergillus flavus* Aflatoxin B1	(1) Spore/mycelial: affects spore development [44]; (2) Cell wall/membrane integrity: changes membrane permeability, leading to the leakage of biomacromolecules and ions [44,45]; changes mitochondrial membrane potential [45]; (3) Metabolic level: inhibits ATPase activity and increases the generation of ROS [44,45]; affects amino acid metabolism [44]; alters ergosterol content, C-sources utilization, and Nor-1 gene expression [45].
*Cleome viscosa* EOs [46] *Aegle marmelos* L. Corrêa EOs [47]	*Candida albicans*	(1) Cell wall/membrane integrity: reduces the chitin levels and interferes with cell wall biosynthesis [46]; binds to ergosterol in the membrane and results in ion permeability increase [47].
Tea tree EOs [48] *Monarda didyma* L. EOs [49]	*Alternaria solani*	(1) Spore/mycelial: affects mycelial growth [48,49]. (2) Metabolic level: increases the peroxidase and phenylalanine ammonialyase activities [48].
*Monarda didyma* L. EOs [49]	*Colletotrichum* sp.	(1) Spore/mycelial: affects mycelial growth [49].

**Table 3:** The release models and release mechanism of EOs microcapsules.

Core Materials	Wall Materials	Microcapsule Method	Release Model	Release Mechanism	References
Coriander EOs	Chitosan/Chitosan-Alginate/	Spray-drying	M_t_/M_∞_ = kt^n^	Anomalous diffusion	[52]
Coriander EOs	Alginate/Chitosan-Inulin	Spray-drying	M_t_/M_∞_ = kt^n^	Diffusion swelling	[52]
Lime EOs	whey protein	Spray-drying	M_t_/M_∞_ = kt^n^	Fickian diffusion	[53]
Lime EOs	whey protein-inulin	Spray-drying	M_t_/M_∞_ = kt^n^	Anomalous diffusion	[53]
Lime EOs	whey protein-oligofructose	Spray-drying	M_t_/M_∞_ = kt^n^	Anomalous diffusion	[53]
Spearmint EOs	Inulin-Gum Arabic	Spray-drying	M_t_/M_∞_ = k_1_t^m^ + k_2_t^2m^	Fickian diffusion	[12]
Juniper berry EOs	Gum arabic/Gum arabic-Maltodextrin/Sodium Alginate	Spray-drying	M_t_/M_∞_ = k_1_t^m^ + k_2_t^2m^	Fickian diffusion	[54]
Tea tree EOs	Polylactic acid/Octenyl succinic anhydride chitosan	Double emulsion and solvent evaporation method	M_t_/M_∞_ = k_1_t^m^ + k_2_t^2m^	Fick diffusion and skeletal dissolution	[55]
Rosemary EOs	Chitosan-Gelatin	Spray drying	M_t_/M_∞_ = kt^n^	Fickian diffusion	[56]
Oregano EOs	Chitosan-decorated Titanium Dioxide	Ion-exchange-mediated self-assembly technique	M_t_/M_∞_ = kt^n^	Fickian diffusion	[57]
Caprylic/capric triglyceride	Melamine-formaldehyde	Crosslinked method	M_t_/M_∞_ = kt	Burst release	[58]
Caprylic/capric triglyceride	Polystyrene	Crosslinked method	M_t_/M_∞_ = kt^n^	Diffusion	[58]
Cinnamon EOs	Persian gum-maltodextrin	Spray-drying	M_t_/M_∞_ = k_1_t^m^ + k_2_t^2m^	Fickian diffusion	[59]
Cinnamon EOs	Persian gum-maltodextrin	Spray-drying	M_t_/M_∞_ = kt^n^	Fickian diffusion	[59]
Lemon EOs	Polyurethane-lignin	A joint method of interfacial polymerization with free radical copolymerization	M_t_/M_∞_ = kt^1/2^	Diffusion	[60]
*Perilla frutescens* L. EOs	Starch sodium octylsuccinate/sodium alginate/chitosan	Coacervation methods	M_t_/M_∞_ = k_1_t^m^ + k_2_t^2m^	Fick diffusion	[61]

Note: In the first-order (M_t_/M_∞_ = kt) and Korsmeyer–Peppas (M_t_/M_∞_ = kt^n^) models, M_t_/M_∞_ is the fraction of drugs released, k is the kinetic constant, t is the release time, and n is the diffusional exponent for drug release. In the Peppas–Sahlin model (M_t_/M_∞_ = k_1_t^m^ + k_2_t^2m^), where the first term of the right-hand side is the Fickian contribution, the second term is the Case-II relaxational contribution. The coefficient m is the purely Fickian diffusion exponent for a device of any geometrical shape which exhibits controllable release, and k_1_ and k_2_ are diffusion and erosion constants, respectively [62].
